# The Genome Conformation As an Integrator of Multi-Omic Data: The Example of Damage Spreading in Cancer

**DOI:** 10.3389/fgene.2016.00194

**Published:** 2016-11-15

**Authors:** Fabio Tordini, Marco Aldinucci, Luciano Milanesi, Pietro Liò, Ivan Merelli

**Affiliations:** ^1^Computer Science Department, University of TorinoTorino, Italy; ^2^Institute of Biomedical Technologies, Italian National Research CouncilMilan, Italy; ^3^Computer Laboratory, University of CambridgeCambridge, UK

**Keywords:** gene functional annotations, chromosome conformation capture, metabolic pathways, epigenetic patterns, gene expression, cancer mutations, multi-layer networks, damage spreading

## Abstract

Publicly available multi-omic databases, in particular if associated with medical annotations, are rich resources with the potential to lead a rapid transition from high-throughput molecular biology experiments to better clinical outcomes for patients. In this work, we propose a model for multi-omic data integration (i.e., genetic variations, gene expression, genome conformation, and epigenetic patterns), which exploits a multi-layer network approach to analyse, visualize, and obtain insights from such biological information, in order to use achieved results at a macroscopic level. Using this representation, we can describe how driver and passenger mutations accumulate during the development of diseases providing, for example, a tool able to characterize the evolution of cancer. Indeed, our test case concerns the MCF-7 breast cancer cell line, before and after the stimulation with estrogen, since many datasets are available for this case study. In particular, the integration of data about cancer mutations, gene functional annotations, genome conformation, epigenetic patterns, gene expression, and metabolic pathways in our multi-layer representation will allow a better interpretation of the mechanisms behind a complex disease such as cancer. Thanks to this multi-layer approach, we focus on the interplay of chromatin conformation and cancer mutations in different pathways, such as metabolic processes, that are very important for tumor development. Working on this model, a variance analysis can be implemented to identify normal variations within each omics and to characterize, by contrast, variations that can be accounted to pathological samples compared to normal ones. This integrative model can be used to identify novel biomarkers and to provide innovative omic-based guidelines for treating many diseases, improving the efficacy of decision trees currently used in clinic.

## Introduction

The application of high-throughput genomic, proteomic, and transcriptomic experiments has gradually become an inevitable component for biomedical science. The reason is that omic data helps the interpretation of the mechanisms that cause the onset of complex diseases. In particular, these data help in cracking the immense complexity of the cancer genome, improving our comprehension of the striking heterogeneity also in histologically similar tumors (Vucic et al., 2012).

Our approach moves from the consideration that during the disease progression some omic processes come before others: typically, mutations are the early factors that influence the onset of the pathology. For example, we are now able to create phylogenetic trees of cancers, describing the accumulation of mutations during time, which transform cells impairing growth constraints and allowing their invasiveness.

But cells can become pathological also in consequence of alterations in the surrounding microenvironment, rather than for mutations in their own DNA, and epigenetics changes reflect these cellular interactions (Maffini et al., 2004). Recent evidences in cancer development point to a crosstalk between these two mechanisms, suggesting that gene mutations have the potential of disrupting several epigenetic patterns and that epigenetic modifications can drive genomic instability and mutagenesis. For example, whole exome sequencing of thousands of human cancer cells revealed unexpected mutations in genes involved in epigenetic mechanisms, and those mutations have the potential to disrupt DNA methylation patterns, histone modifications and nucleosome positioning (Coppedè et al., 2014).

**Figure 1:**
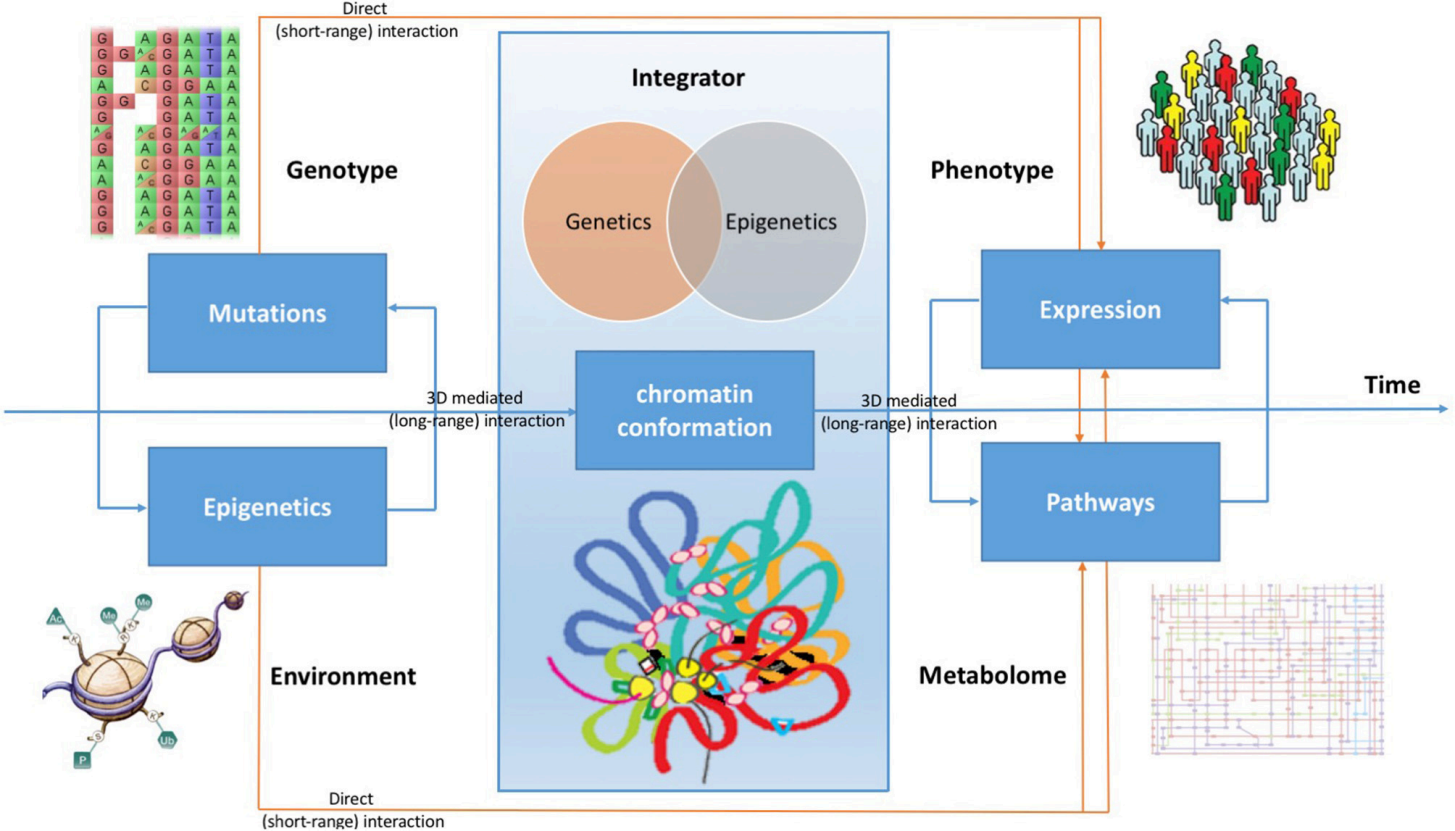
**Chromatin conformation as an integrator of omic signals**. Cancer can evolve by accumulating many mutations, which impact on the expression profile of cells, but also collecting from the microenvironment stimuli that boost cell transition to tumor, causing genomic instability. Beside having direct effects on gene expression, therefore influencing metabolic pathways, all these signals are integrated by the conformation of the DNA in the nucleus. During the development of the disease, changes in long-range genome interactions impact on cell's regulatory patterns. By combining all these effects, the expression profile of cells acquires the typical hallmarks of cancer, changing its metabolic characteristics and modifying its molecular pathways.

Beside having direct effects on gene expression, therefore influencing metabolic pathways, all these pathogenic mechanisms play a role in reshaping the 3D chromatin conformation, modifying long-range interactions in the genome. Indeed, there are evidences that DNA topological changes occurring during the disease development support the conversion of cells to a pathological state (Jäger et al., 2015; Du et al., 2016). Considering cancer, although chromatin conformation changes are poorly understood during the tumor development, it is possible to demonstrate that oncogenic transcription-factors overexpression is associated with global, reproducible, and functionally coherent changes in the chromatin organization (Rickman et al., 2012; Taberlay et al., 2016). A better comprehension of chromatin conformation modifications can be achieved by integrating data concerning genetic variations, genome conformation, gene expression, epigenetic patterns, gene functional regulation, and metabolic pathways (see Figure 1).

Evidences of the 3D genome conformation role in regulating and maintaining cellular functions are continuously emerging (Lieberman-Aiden et al., 2009; Dong et al., 2010; Schoenfelder et al., 2010; Gerstein et al., 2012; Kalhor et al., 2012; Lan et al., 2012; Shen et al., 2012; Homouz and Kudlicki, 2013; Jin et al., 2013; Papantonis and Cook, 2013; Babaei et al., 2015). For example, pathological genome organizations can be associated with many diseases, such as muscular dystrophy (Petrov et al., 2008) and Rett syndrome (Horike et al., 2005). The genome folding plays a critical role also in chromosomal rearrangements that occur during the creation of the antibody repertoire as part of the immunological development (Merelli et al., 2010; Zhang et al., 2012). Moreover, insertion sites selected by retroviruses (and retroviral vectors in gene therapy) to inject their exogenous genomic material in the host genome are largely influenced by the 3D organization of chromosomes (Biasco et al., 2011).

For example, Genome-Wide Association Studies (GWAS) have identified more than 70 common single nucleotide polymorphisms (SNPs) that are associated with the breast cancer risk (Michailidou et al., 2013). However, the vast majority of these SNPs lie in noncoding regions of the genome and their interpretation is difficult (Merelli et al., 2013a; Kel et al., 2016). To test whether SNPs regulate their target genes through long-range chromatin interactions, capture-based sequencing technology have been used to investigate possible *cis*-interactions at different cancer risk loci (Jäger et al., 2015; Du et al., 2016), finding very interesting results in this sense. More generally, the association between chromosome conformation and epigenetic patterns is under investigation, by comparing normal and cancer tissues. The organization of the chromatin in the nucleus can be itself a biomarker (Babu and Fullwood, 2015), since specific reorganizations of the genome in the nucleus can characterize the early onset of tumors, as it has been demonstrated for breast cancer (Meaburn et al., 2009).

In the past, researchers were able to study the position of the DNA in the nucleus through microscopy, using light reflecting antibodies able to recognize specific genes. However, since 2002, a technology called Chromosome Conformation Capture (3C) permits to stabilize the DNA conformation before sequencing the genome, allowing the identification of sequences that are close to each other in the 3D space of the nucleus. 3C techniques are producing a huge amount of data concerning the conformation of our genome and important studies have already been accomplished to investigate how the chromatin is organized into domains of co-ordinately regulated enhancers and promoters (Shen et al., 2012). However, while there is a rapid growth in data production and improvements in experimental protocols (Barutcu et al., 2016), suitable computational approaches are still required to turn these data into real clinical knowledge.

Experimentally, 3C consists in stabilizing protein-mediated DNA interactions through formaldehyde. The cross-linked genome is enzymatically digested and the resulting pieces of genomic DNA are ligated. This approach generates new binary connections, relying on the spatial proximity of chromosomes in the nucleus, which can be sequenced as paired-end reads. Coupling 3C with massive parallel sequencing allows the study of the genome folding in the nucleus at unprecedented resolution. High Throughput 3C methods (HT-3C), such as Hi-C and chromatin interaction analysis by paired-end tag sequencing (ChIA-PET), have made it possible to generate genome-wide data about interactions between chromatin segments at Kb–Mb resolutions, thus opening the way for studying the 3D genome conformation at these scales.

Although HT-3C can provide very interesting results, this experimental procedure is far from being perfect: not all the established contacts are real and not all the effective contacts are identified. Even more complex is to interpret data, which means to understand why a contact is there, trying to explain the association between two pieces of chromatin (and their corresponding genomic features) that are near each other inside the nucleus. Are these genes co-localized because they are involved in the same metabolic pathway? Are they controlled by the same genetic and epigenetic regulation? How mutations impact on the conformation of the DNA in the nucleus? How do epigenetic factors modulate the genome conformation? Why are these genes close to each other in some cells, while they are distant in others? Is the activity of these genes effectively correlated to their positions? And, the more important: how do changes in the genome conformation correlate with cancer?

To answer these questions, there are few computational and statistical solutions at the moment. The most popular approach to HT-3C data analysis relies on contact maps, which are matrices of pairwise contact frequencies in the genome. This representation, despite being simple from a mathematical point of view, makes difficult to capture the complexity of the nuclear organization, since it only provides a description of binary interactions, preventing the creation of a metric about the distances of different genomic segments. To improve the analysis of HT-3C data, graph-based approaches have been proposed, such as CytoHi-C (Shavit, 2013) and Homer (Heinz et al., 2010), which both rely on Cytoscape for network analysis.

In a previous work we developed NuChart (Merelli et al., 2013b, 2015), an R package that elaborates Hi-C information to provide a systems biology oriented, gene-centric view of the three-dimensional organization of the DNA in the nucleus. In this paper, we want to improve the graph representation of Hi-C data discussed in previous works, proposing a multi-level approach able to integrate different omics using multi-level networks. This representation can describe in a single model the topology of the DNA in the nucleus, epigenetic profiles, cancer mutations, gene functional relations, gene expression, and metabolic pathways, creating an integrative environment that is still lacking in multi-omic data analysis.

We will show how this modeling approach is able to characterize the correlation between the function of some genes, their spatial distribution and the progression of complex diseases, such as cancer. Extending gene network representations to multi-layer models will improve their descriptive power, in order to identify the mechanisms behind gene co-localization, co-expression, and co-regulation. Ideally, by defining appropriate confidence intervals for each different biological feature, we will be able to introduce the concept of variance analysis for omic sciences, allowing multi-omic data to be really translational, since this information can be used to improve decisions in the treatment of patients and also to ameliorate the predictive power of survival curves. In particular, we will test this framework on data from a breast cancer cell line, to describe the spatial, functional and regulatory differences in two distinct conditions.

## Materials and methods

This paper shows how a multi-layer approach for the integration of different omic datasets, representing multiple aspects of the pathology evolution, can be used to model and analyse heterogeneous data, improving medical treatments and achieving better predictions about the disease outcomes. Several bioinformatic tools and network theory concepts have been combined in a unique framework to achieve an integrative multi-layer representation of multi-omic data, which are presented below.

### Biological layers

Although different combinations of networks and scalar data are possible, the framework we propose is three-layered. The first layer is composed by data about mutations, mapped on networks representing gene-to-gene functional similarities, relying on the Biological Process (BP) domain of the Gene Ontology (GO) (Peng et al., 2014). In this way, we want to verify if disease related genomic variations are correlated with specific biological functions, with a particular interest in metabolism, in order to characterize critical genes.

The second layer models the genome conformation, since it represents Hi-C data processed with NuChart (Merelli et al., 2013b, 2015), on which epigenetic profiles are mapped as features of the vertices. The choice of mapping epigenetic patterns on nuclear maps is oriented at studying specific chromatin profiles that influence the final confirmation of the DNA in the nucleus, highlighting regulatory patterns.

**Figure 2:**
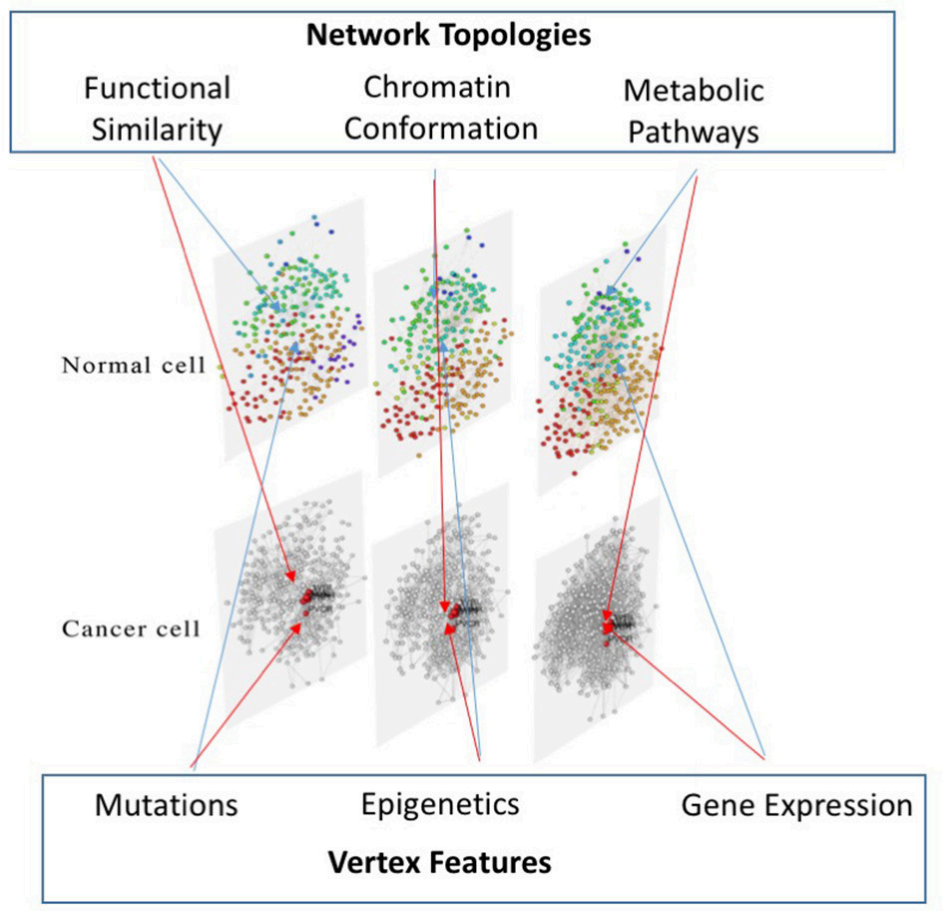
**Multi-layer approach to multi-omic data integration**. Our approach consists of a three-layered model to compare cells in different conditions: the first layer represents the functional similarity of genes (according to the BP domain of GO) on which mutation data from the COSMIC database are mapped; the second layer represents the conformation of the chromatin in the nucleus (according to Hi-C data analyzed with NuChart) on which epigenetic information of cells are mapped, relying on MeDIP-seq experiments; the third layer represents a protein-protein interaction network (according to the STRING database) on which gene expression data are mapped.

The third layer is created mapping gene expression profiles on protein-protein interaction data, as reported in STRING (Jensen et al., 2009). Therefore, this layer is mainly devoted at identifying up and down regulated pathways in different experimental conditions. To achieve the multi-layer model we use MuxViz (De Domenico et al., 2015), which allows to integrate these three layers and to apply inter-layer clustering algorithms (see Figure 2) as well as other diagnostics and modeling tools.

#### Mutations and functional information

The GO initiative provides rich information and a convenient way to study gene functional similarity, which has been successfully used in various applications. However, the existing GO-based gene function similarity measurements are quite difficult to use and only few tools are able to compute metrics taking into account the whole ontology structure. To define a gene functional topology according to GO, a novel integrative measure (and the related analysis tool) called *InteGO2* has been used, which automatically selects the appropriate seed measures and then integrates them using a meta-heuristic search method (Peng et al., 2014). By using this approach, we obtain the first layer of our representation that describes the functional similarity of genes, on which information about mutations of disease associated genes can be mapped.

Many different databases are available to download data about SNPs involved in specific diseases. For example, one of the most used databases for genomic variations involved in tumors is COSMIC, the Catalog Of Somatic Mutations In Cancer (Forbes et al., 2015), because it is the largest and most comprehensive resource for exploring the impact of driver and passenger mutations in human cancers. The latest release describes two millions of manually curated point mutations in over one million tumor samples and across most of the human genes.

#### Hi-C and epigenetics data

The second layer of our model describes the genome conformation and mostly relies on Hi-C data. As introduced, this method combines Next-Generation Sequencing (NGS) and 3C, a technique in which DNA (together with the proteins that coordinate the chromatin conformation) is cross-linked with formaldehyde, enzymatically fragmented, and re-ligated relying on its physical proximity in the nucleus. From the bioinformatics point of view, chromatin conformation data have been analyzed using NuChart (Merelli et al., 2013b, 2015), a complete suite of tools for the analysis of Hi-C experiments using a gene-centric point of view, to provide a map on which other omic information can be mapped.

In order to complete the Hi-C layer, it can be desired to map epigenetic data on the neighborhood graph of a gene, such as methylation and histone modification. Typically, the experiments used to study these epigenetic patterns rely on chromatin immunoprecipitation sequencing (ChIP-seq), a method used to analyse protein interactions with DNA. A possible choice is to use data achieved through Methylated DNA immunoprecipitation sequencing (MeDIP-seq), a large-scale purification technique used to enrich for methylated DNA sequences, which relies on isolating methylated DNA fragments via an antibody raised against 5-methylcytosine followed by massive parallel sequencing.

#### Interaction and expression data

Protein-protein interaction networks are an important ingredient for the system-level understanding of cellular processes, and omic data analysis heavily depends on high quality knowledge-base of pathway maps. A very useful database in this context is STRING (Search Tool for the Retrieval of Interacting Genes/Proteins) (Jensen et al., 2009). The STRING database contains information from numerous sources, including experimental data, computational prediction methods, and public text collections.

We use the information available in STRING to define the topology of the third layer of our model, which represents the phenotype, in terms of gene expression and metabolic pathways, achieved by pathological cells according to the modifications of the genome conformation. To this end, the obvious choice is to map on this layer the expression of genes, in order to highlight possible correlations between gene co-expression, co-localization, and co-regulations in cancer cells.

#### Bioinformatics pipeline

From the bioinformatic point of view, the multi-layer model is the result of a pipeline that encompasses a number of steps in which the tools highlighted above have been employed. The whole process starts by identifying some genes of interest, called seed genes.

We start by computing the neighborhood graph for the seed genes using NuChart, working on the raw sequencing data, usually downloaded from the NCBI Short Read Archive (SRA) database, of the appropriate Hi-C experiment (since, in our example, we need raw sequencing data to create our neighborhood graphs). Once the Hi-C graph has been constructed (considering a user defined distance from the seed gene), we skim the edges list, taking only edges with weight (i.e., probability of existence) greater than a specific threshold (default *p* <0.05). From this list of edges, we retrieve all unique genes that play a part in the current network, and use this list as input for the InteGO2 web application. With InteGO2 we can compute the gene-to-gene functional similarity, which is returned as a network that links genes that are functionally similar. At the same way, we build the protein-protein interaction network for the seed genes using the STRING search tool (considering a user defined distance from the seed gene).

The next step is to map genomic data on the achieved layers: as already explained, we map epigenetic data on the Hi-C layer, expression data on the STRING layer, and gene mutations on the InteGO2 layer, although other combinations are possible.

Concerning epigenetics, experimental results can be typically downloaded from the NCBI Gene Expression Omnibus (GEO) database, where pre-processed ChIP-Seq data are publicly available (in our example, MeDIP-seq data were accessible for the same cells of the selected Hi-C experiments). The pre-processed output can be usually downloaded as text files, which contain enriched peaks (default *p* <0.05), usually called using MACS (Zhang et al., 2008). While rendering graphs, we use the average value of the considered epigenetic mark, taking into account all the genes playing a role in the Hi-C layer, as a threshold to differentiate the graphical look of the nodes and coloring the vertices accordingly. Moreover, the logarithm of the normalized epigenetic values is used to determine the graphical size of the node.

A rather similar approach has been used for mapping expression data and mutations on the STRING and InteGO2 layers, respectively. Pre-processed expression data can be freely downloaded both from the EMBL-EBI ArrayExpress database (in our example, gene expression data were accessible for cells in the same conditions and at the same time points of the selected Hi-C experiments) as text files, which contains differential expressed genes (default *p* < 0.05). When rendering the graph, we compute the average expression value of all the genes in the STRING layer, and we then use this value as a threshold for choosing the color of the vertices, as explained in the previous paragraph, while the logarithm of the expression value determines the graphical size of the node.

At the same way, mutations are mapped on the InteGO2 layer: the mutation dataset is downloaded from a reference database (in our example, from the COSMIC catalog), according to the pathology in analysis, which typically describes, for each gene, the number of analyzed samples carrying mutations in relation to the number of tested samples. We use this number for determining both color and size of the nodes in the GO-based layer, according to the protocol described above.

### Multi-layer integration

A network representation is useful for describing the structure of a large variety of complex systems, although most real frameworks have multiple subsystems and layers of connectivity. Achieving a deep understanding of such systems necessitates the generalization of the traditional network theory, and the huge amount of data generated by NGS is a very interesting ground to test increasingly complex frameworks for network analysis (Forbes et al., 2015).

#### Multi-layer formalization

When we refer to multi-layer networks we adhere to the definition provided in (Kivelä et al., 2014), where interconnected systems are described as networks where many, or even all the actors of the system have a counterpart in each layer, so that one can associate a vector of states to each actor. Multi-layer networks can encode much richer information than the individual layers separately. This, in turn, provides a suitable framework for versatile analyses that are widely used to reveal multi-layer community structures, to identify node clusters, and to compute correlation statistics between them.

From the mathematical point of view, adjacency matrices are useful to describe traditional single-layer networks, but such representations are insufficient for the analysis and description of interconnected networks. One must therefore develop a more general mathematical framework to cope with the challenges posed by multi-layer complex systems. The principal theoretical foundations for a wide variety of multi-layer networks have been laid out in Kivelä et al. (2014), in an attempt to present a unifying framework to treat both traditional single-layer networks and a variety of complex networks successfully.

In particular, a graph (i.e., a single-layer network) is a tuple *G* = (*V, E, w*), where *V* is the set of nodes (or vertices) and *E* ⊆ *V* × *V* is the set of edges that connect pairs of nodes according to some type of relationship. *w* is a function *w: E* → ℝ^+^ that assigns a *weight* to the edge, thus qualifying the relation between two nodes. We can thus define *e*_*i, j*_ as the edge that connects node *i* to node *j*, with *w*_*i, j*_ being the weight of the edge that connects node *i* and node *j*. If there is an edge between a pair of nodes (*e*_*i, j*_, *e*∈*E*), then those nodes are *adjacent* to each other. This edge is *incident* to each of the two nodes, and two edges that are incident to the same node are also said to be *incident* to each other.

In multi-layer networks, we will use the term *adjacency* to describe a connection between a pair of node-layers, and the term *incidence* to describe two (or more) edges that connect a node-layer pair. Follows that two edges that are incident to the same node-layer pair are also incident to each other. By assembling a set of layers *L* using a Cartesian product *L*_1_ × … × *L*_*d*_, we can indicate whether a node is present in a given layer. To do so, we first construct a set *V* × *L*_1_ × … × *L*_*d*_ of all the combinations of a node and a layer, and then define a subset *V*_*M*_ ⊆ *V* × L_1_ × … × L_*d*_ that contains only the node-layer combinations in which a node is present in the corresponding layer. In multi-layer networks we also need to specify the starting and ending layers for each edge: in this perspective, *E*_*M*_ is the set of pairs of all possible combinations of nodes and layers: *E*_*M*_ ⊆ *V*_*M*_ × *V*_*M*_. Using the components set up above, a multi-layer network is defined as a quadruplet *M* = (*V*_*M*_, *E*_*M*_, *V, L*).

#### Multi-layer representation

MuxViz is a framework designed for the analysis and visualization of multi-layer networks. It allows an interactive visualization and exploration of graphs where nodes exhibit multiple relationships simultaneously, on different layers (De Domenico et al., 2013). By combining two standard force-directed algorithms, it determines the positions of nodes in each layer, and project them to an aggregated network obtained by summing the corresponding entries of the adjacency matrices of the individual layers. Specifically, first the *Fruchterman–Reingold* algorithm is applied to the *aggregated* network, and then the output of this algorithm is used as a seed layout for the *Kamada–Kawai* algorithm to achieve a better spatial separation of nodes in the final layout (De Domenico et al., 2013).

MuxViz provides a generalization of several important network descriptors—including degree centrality, clustering coefficients, eigenvector centrality, etc.—by means of tensor formalisms and higher-order tensor algebra. Tensors provide a convenient mathematical representation for generalizing ordinary static networks, and permit to encapsulate complicated sets of relationships that can also change in time. MuxViz can also derive the aggregated network from the interconnected structure, where the edges between two actors are summed up across all layers. The aggregated layer puts in evidence how topological descriptors of interconnected networks differ from the ones corresponding to their aggregated graphs.

#### Centrality measures and correlations

We defined above our single-layer network as tuples *G* = (*V, E, w*), resulting in undirected weighted graphs. The identification of the most “important” nodes in a system has great importance in network characterization. The most intuitive topological measure of centrality is given by the *degree* of the nodes: more connected nodes are more central. The degree of a node *d*_*i*_ is defined as the sum of its incident edges:

$$d_{i}=\Sigma_{j\;}e_{i,j},\;\;e\in E,\;(i,j)\in V$$

We can take into account the degree of each node in each layer *L* of our multi-layer model, as well as the degree of the nodes in the aggregate layer. However, *more* is not necessarily *better*: the weights of the edges differentiate them according to the relationship that ties the nodes. In our case, nodes relationships vary according to the information encoded in the graph—functional similarity, spatial proximity and physical interaction, respectively. Taking into account the weight of the edges we are able to analyse the *strength* of each node *s*_*i*_, that is defined as the sum of weights attached to the edges belonging to a node:

$$s_{i}=\Sigma_{j\;}e_{i,j}w_{i,j},\;\;e\in E,\;(i,j)\in V$$

This quantity measures the strength of vertices in terms of the total weight of their connections. In each layer *L* of our multi-layer model, connections reflect a particular relationship quantified with values ranging between 0 and 1, thus reporting the probability for that relationship to exist. With MuxViz we can compute the strength of each node in each layer, separately, plus the strength of the nodes in the aggregate layer, resulting in a natural measure of the importance of a vertex *i* in our model.

Working on the resulting graphs we can apply some centrality statistics to identify those “important” vertices that are likely to be highly influential for the dynamics of the described mode, such as cancer super-spreaders. By using the tensorial calculation introduced above, MuxViz permits to extend well known statistics for centrality analysis in multi-layer networks. Among the others, the *HITS* centrality (Hyperlink-Induced Topic Search) is a link analysis algorithm that was introduced to rank websites in relation to their importance for users. This approach considers two different descriptors for each node, namely *hub* and *authority*: in the context of the World Wide Web, certain web pages that point to an important page, generally also point to other important pages, building a structure similar to a bipartite topology where relevant pages—i.e., authorities—are pointed by special web pages—i.e., hubs—which are not actually authoritative in the information that they held, but directly connect to many other authoritative pages. Follows that nodes with high authority centrality are linked by nodes with high hub centrality, while very influential hubs point to nodes which are very authoritative.

With MuxViz we can also calculate a measure of correlation (e.g., *Pearson* or *Spearman* correlation) between each pair of descriptors, to obtain a set of pairwise distances and measure the similarity between layers. The *inter-layer assortativity* module computes the Pearson correlation between the degree (and strength) of nodes and their counterparts in other layers, for all pairs of layers, so as to measure the linear correlation between two variables, *X* and *Y*, returning a value in the range [+1, −1], where 1 is total positive correlation, 0 is no correlation, and −1 is total negative correlation. An alternative correlation analysis is the Spearman correlation, that is again a nonparametric measure of statistical dependence between two variables, but is recommended when the assumptions underlying a Pearson test are not satisfied. This is another measure of similarity between layers.

## The breast cancer case study

To discuss our approach on a specific test case for the characterization of damage spreading in cancer, we used data from the MCF-7 breast cancer cell line, for which many different omic experiments are available. MCF-7 is a breast cancer cell line isolated in 1970 from a 69-year-old Caucasian woman, which retained several characteristics of differentiated mammary epithelium, including the ability to process estradiol via cytoplasmic estrogen receptors and the capability of forming domes.

Our primary data source is the work of Mourad et al. (2014), in which a time series Hi-C experiment has been discussed as a promising methodology for a better understanding of the chromatin conformation global dynamics and its link with gene regulation (GEO:GSE51687). This time series corresponds to different sampling performed after cell treatment with *17*β*-estradiol* (E2), the primary female sex hormone, which is responsible for the development and regulation of the female reproductive system and secondary sex characteristics. Estrogen stimulation is an important factor in human breast cancer cell growth and development, since it leads to genome structure reconfiguration, thereby disrupting the originally chromatin structures (Rao et al., 2014).

More precisely, we considered MCF-7 chromatin conformation experiments performed by Mourad et al. (2014) at time point 0 and 4 h after 7 × 10^−8^ M E2 stimulation. These data have been analyzed with NuChart and only edges having high statistical significance (*p* < 0.05) have been retained in the graphs. For gene expression, we used MCF-7 time-series data after E2 stimulation from Cicatiello et al. (2010), considering time points 0 and 4 h after 5 × 10^−8^ E2 stimulation. These time points are shared between Hi-C and gene expression experiments (AE:E-TABM-742) and only differential transcribed genes (*p* < 0.05) have been considered. From the epigenetic point of view, many different aspects play an important role in cancer evolution. Taking into account publicly available datasets, we decided to integrate MeDIP-seq data concerning methylation in MCF-7 from Hsu et al. (2010). These data are from MCF-7 cells that were not stimulated with E2, since methylation patterns are mostly static in response to E2 within the 24 h (Ross-Innes et al., 2011; ENCODE Project Consortium, 2012; Putnik et al., 2012), and only peaks identified with high confidence (*p* < 0.05) have been considered in the model (GEO:GSE21068).

**Figure 3:**
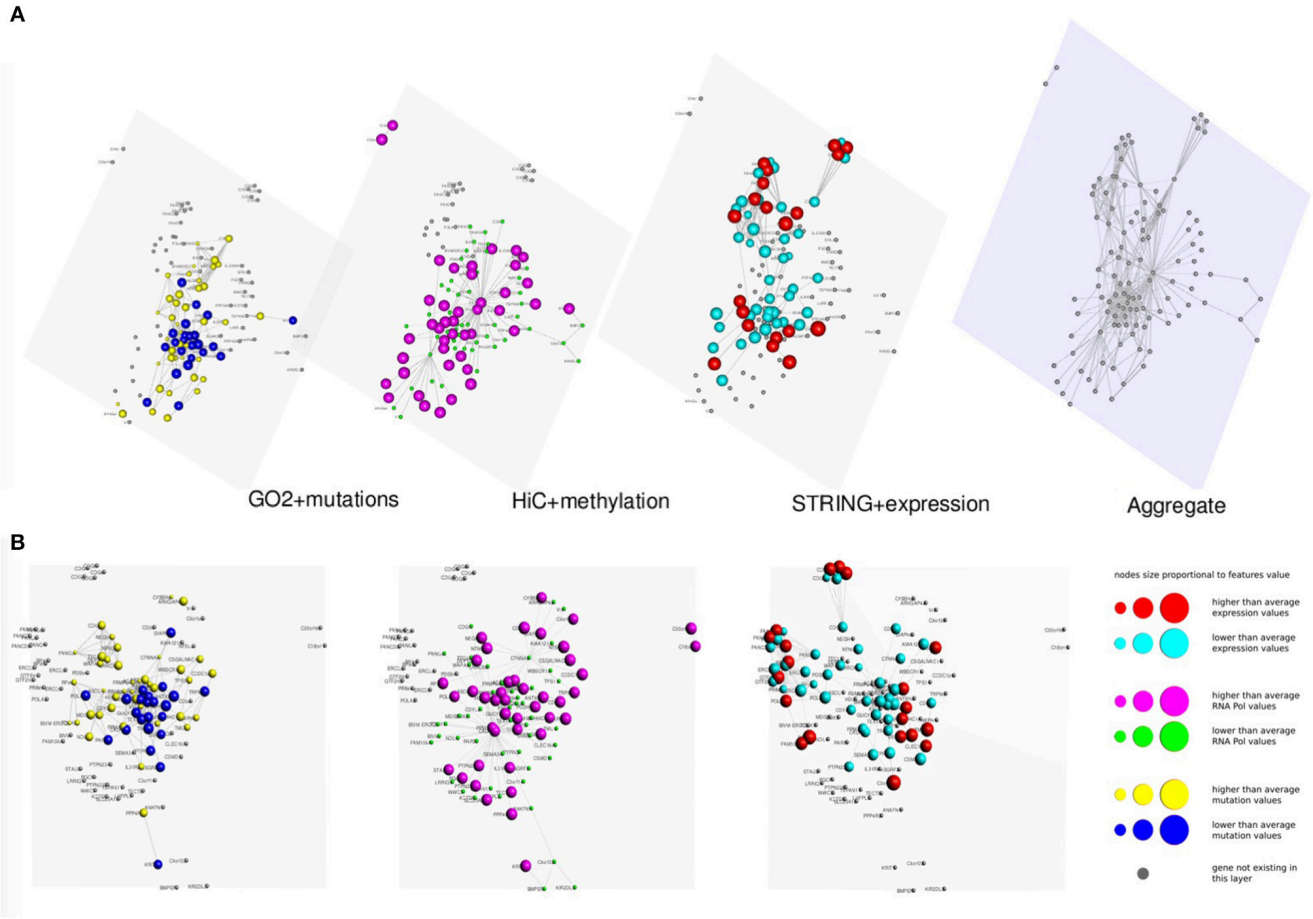
**Multi-layer graph for the breast cancer cell line MCF-7 at time point 0 h, before E2 stimulation, considering ESR1 as seed gene. (A)** represents the stacked multilayer (interlayer edges have been removed for readability), while **(B)** shows each single layer separately. In both panels, from left to right, the first layer represents the functional similarity of genes relying on the BP domain of GO, as computed by InteGO2. On this network, breast cancer mutation data are mapped, according to the COSMIC database (colors and dimensions of nodes indicate the number of samples with mutations in the COSMIC dataset for each gene). The second layer represents the conformation of the chromatin in the nucleus, according to Hi-C experiments analyzed using NuChart. Vertices are colored according to the methylation of genes, relying on MeDIP-seq experiments. The third layer represents a protein-protein interaction network according to the data reported in the STRING database. Gene expression data have been mapped on this graph and vertices are represented accordingly. Please note that in each layer genes are mapped always in the same positions, according to the representation achieved in the fourth level of **(A)**, in which all genes are aggregated and the Fruchterman–Reingold is performed to achieve the optimal distribution of vertices.

**Figure 4:**
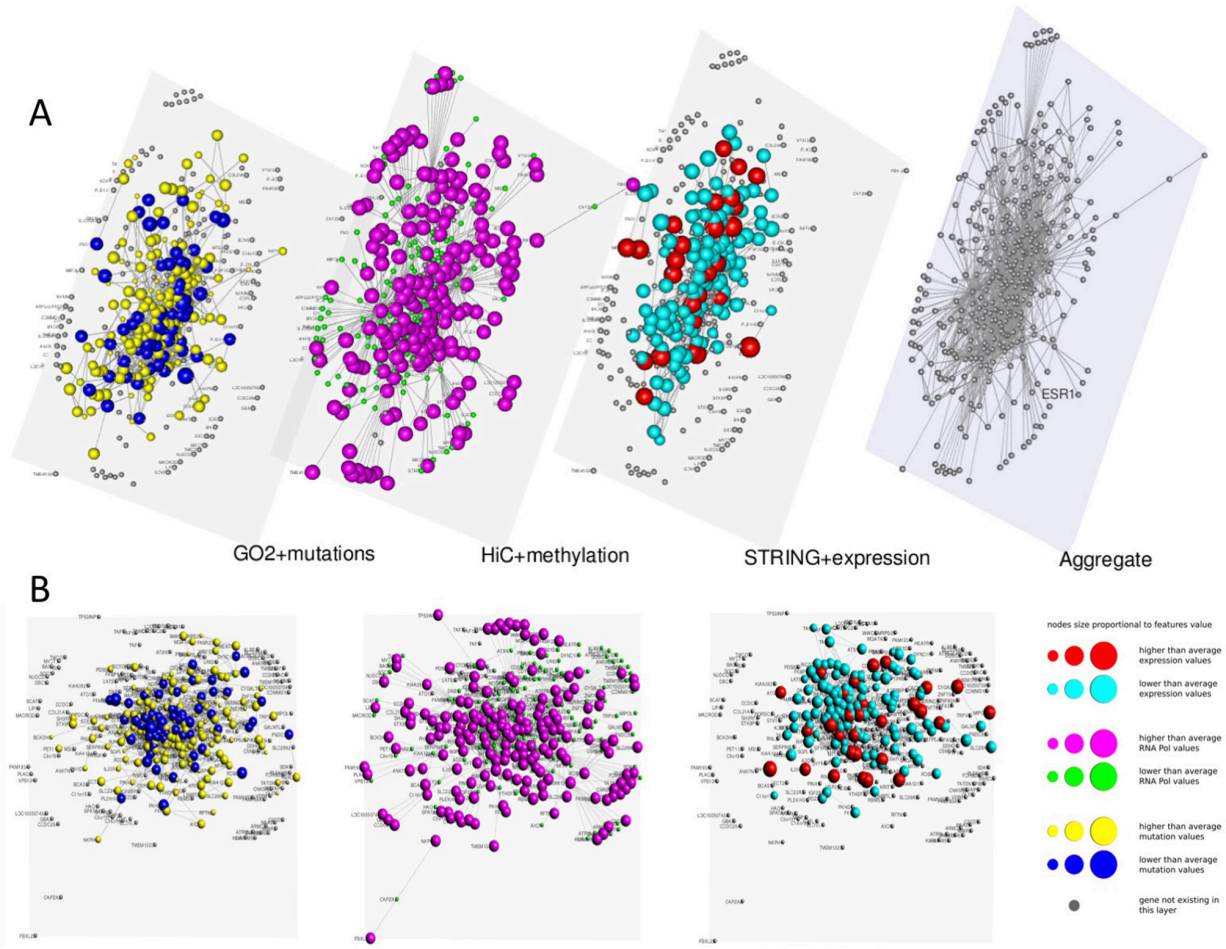
**Multi-layer graph for the breast cancer cell line MCF-7 at time point 4 h after E2 stimulation, considering ESR1 as seed gene. (A)** represents the stacked multilayer (interlayer edges have been removed for readability), while **(B)** shows each single layer separately. In both panels, from left to right, the first layer represents the functional similarity of genes relying on the BP domain of GO, as computed by InteGO2. On this network, breast cancer mutation data are mapped, according to the COSMIC database (colors and dimensions of nodes indicate the number of samples with mutations in the COSMIC dataset for each gene). The second layer represents the conformation of the chromatin in the nucleus, according to Hi-C experiments analyzed using NuChart. Vertices are colored according to the methylayion of genes, relying on MeDIP-seq experiments. The third layer represents a protein-protein interaction network according to the data reported in the STRING database. Gene expression data have been mapped on this graph and vertices are represented accordingly. Please note that in each layer genes are mapped always in the same positions, according to the representation achieved in the fourth level of **(A)**, in which all genes are aggregated and the Fruchterman–Reingold is performed to achieve the optimal distribution of vertices.

In Figures 3, 4 we present two multi-layer graphs, as achieved with MuxViz, for the breast cancer cell line MCF-7 at time point 0 and 4 h after E2 stimulation (see Supplementary Tables 1, 2 for the full gene lists). In particular, these graphs have been generated using the Estrogen Receptor 1 (ESR1) as seed gene, which a key regulator for the physiological growth and differentiation of the mammary gland, but also a key element for the malignant progression of breast cancer. Once activated by estrogen, the ESR1 is able to translocate into the nucleus and bind to DNA to regulate the activity of different genes, which makes this protein particularly interesting from the chromosome conformation point of view. Using ESR1 as input, we identified its neighborhood genes, according to the available Hi-C experiments. At the same time, we generated the other layers using InteGO2 and STRING. The fourth level in Figures 3, 4 represents the aggregate layer, which shows the whole gene set staged in the other three layers (since, as discussed above, they can present different genes, due to the different connections that can be generated using the seed genes as input of the different tools). The colors and dimensions of vertices are plotted according to the scalar values assigned to each node, representing the different multi-omic features, reported in log scale (see the method section for details). In particular, on the Hi-C layer we mapped gene methylation, on the STRING layer we mapped gene expression, and on the gene functional layer we mapped mutations as retrieved from the COSMIC database.

As it can be seen from the comparison between Figures 3, 4, after the E2 stimulation the genes inside the nucleus achieve a closer conformation, becoming denser around the seed gene ESR1. This gene also changes its expression, since after stimulation it expression doubles, which is somehow expected considering its role in the malignant progression of breast cancer. However, the most evident variation is the number of mutations that appear in the network after the stimulation, which testify for the amount of mutated genes activated by E2. This evidence highlights how variations can diffuse around few critical mutations, increasing damage spreading in cancer cells.

More generally, relying on the results achieved genome wide, this study demonstrates the role of the estrogen on the global organization of the genome and its link with gene regulation in cancer. After E2 stimulation the network shows a more centralized configuration, and methylation patterns seems anti-correlated to active genes, as expected. Besides augmenting the number of long-range interactions, E2 induces a dynamic mechanism of global chromatin reorganization. More specifically, gene-rich chromosomes as well as areas of open and highly transcribed chromatins are rearranged to a greater spatial proximity.

On the other hand, we see a substantial independence between gene expression and gene co-localization, which can be partially explained with a general deregulation of physiological pathways. The impression is that after the E2 stimulation cells are forced to increase their activity, which results in a generalized growth of gene expression. From these results, we can conclude that E2 induces a higher spatial compartmentalization of genes, with a wide activation of genes in open chromatin regions.

## Results

**Figure 5:**
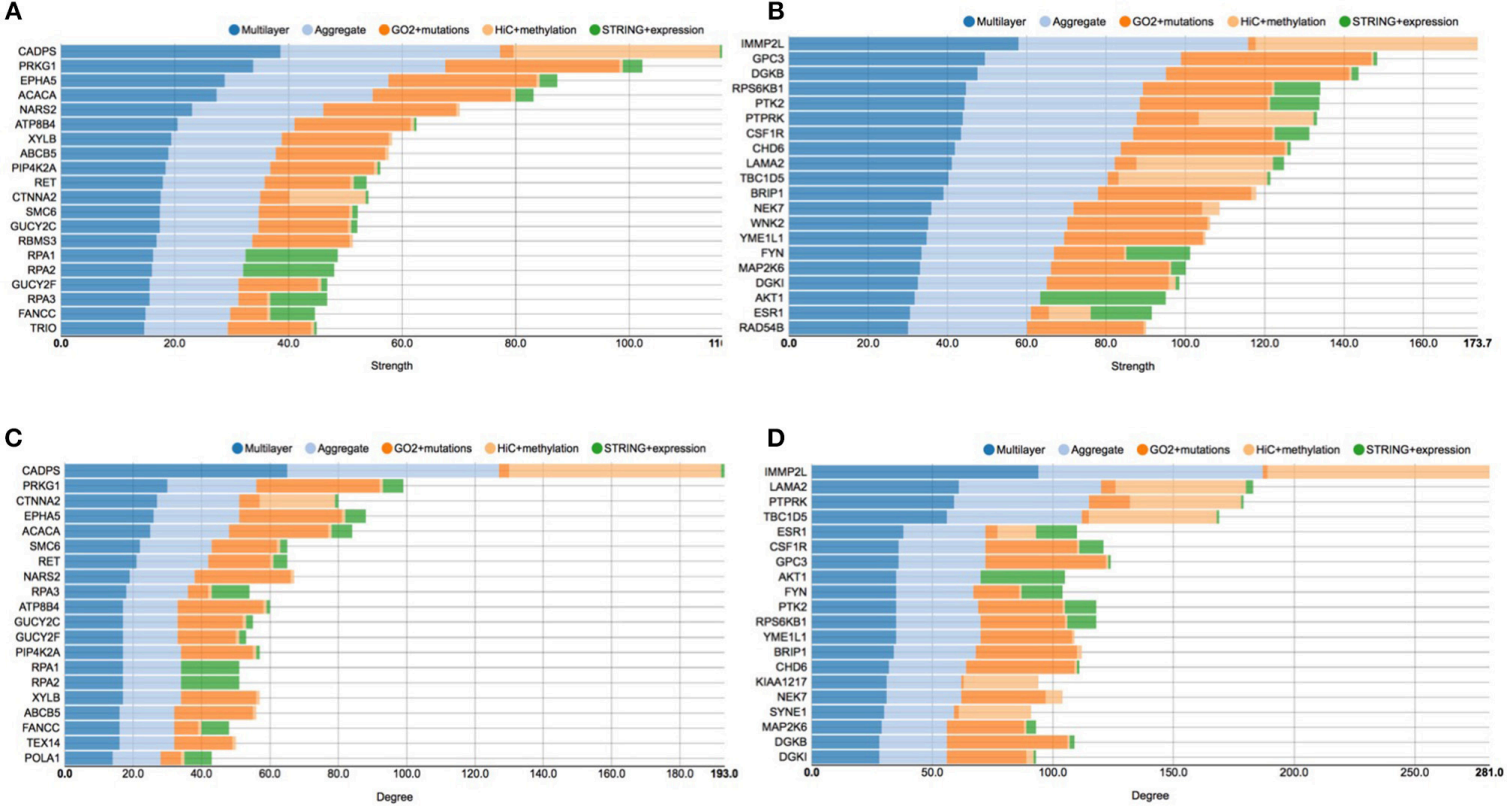
**Stacked histogram plots of centrality measures**. This figure shows the top 20 genes considering the strength and degree of the nodes in the graphs before and after E2 stimulation. Each grouped histogram represents each single layer separately, the aggregate layer (the layer with all the nodes represented in the other layers) and the whole multi-layer model. In particular, **(A)** shows the top 20 genes considering the strength of the nodes in the graphs before the stimulation; **(B)** shows the top 20 genes considering the strength of the nodes in the graphs after the stimulation; **(C)** shows the top 20 genes considering the degree of the nodes in the graphs before the stimulation; **(D)** shows the top 20 genes considering the degree of the nodes in the graphs after the stimulation.

The first analysis we performed on the achieved multi-layer graphs was to compute the distribution of the strength and degree of the graph nodes—which identify genes or proteins, depending on the considered layer—in order to identify the most important genomic players before and after the E2 stimulation. To this end, Figure 5 proposes four charts displaying the top 20 genes in function of node strength and degree. Stacked bars represent each single layer separately (GO functional annotations, chromosome conformation and protein-protein interactions), the aggregate layer (the layer with all the nodes represented in the other layers) and the whole multi-layer model.

In particular, looking at Figure 5A shows the top 20 genes considering the strength of the nodes in the graph before the stimulation, which should be compared to Figure 5B, showing the top 20 genes considering the strength of the nodes in the graph after the stimulation. At the same way, Figure 5C shows the top 20 genes considering the degree of the nodes in the graph before the stimulation, which should be compared to Figure 5D, showing the top 20 genes considering the degree of the nodes in the graphs after the stimulation.

CADPS is the gene showing the highest strength and degree before E2 the stimulation, with a considerable margin on the other nodes. This gene encodes an endocrine-specific cytosolic and peripheral membrane protein required for the Ca^2+^-regulated exocytosis of secretory vesicles. CADPS is down-regulated in many types of tumors, although it has not been reported as associated to breast cancer (Mosca et al., 2010). In the pre-stimulus multi-layer model, we can see a similar distribution of genes in the top list of node strength and degree, since 18 out of 20 genes are shared between the two lists.

According to COSMIC (Forbes et al., 2015), other interesting cancer related genes are present in these top lists, such as EPHA5, which belongs to the ephrin receptor subfamily of the protein-tyrosine kinase family and PIP4K2B, an enzyme that catalyzes phosphorylation, showing kinase activities. More precisely, EPHA5 is implicated in mediating developmental events and plays a critical role in the regulation of carcinogenesis, since it has been demonstrated to be a promoter of methylation in breast cancer (Li et al., 2015). PIP4K2B has been linked to the regulation of gene transcription, to TP53 and AKT activation, and to the regulation of cellular reactive oxygen accumulation. Low PIP4K2B expression associates with increased tumor size and distant metastasis, whereas high PIP4K2B expression strongly associates with ERBB2 expression (Keune et al., 2013).

Considering pathways, we can see genes related to *DNA replication*, such as POLA1 RPA1, RPA2, RAP3, SMC6 (*p* < 4.1e^−8^), which all share a high number of edges in the protein-protein interaction layer and belong to the neighborhood of ESR1, although they do not present many mutations. Moreover, we can see proteins from the *protein phosphorylation* pathway, GUCY2C, GUCY2F, PRKG1, RET, TEX14 (*p* < 1.5e^−3^), which are involved in AKT regulation and signaling during tumorigenesis (Lin et al., 2010).

After the E2 stimulation, at the top of both the node degree and strength lists we find IMMP2L. This gene encodes an inner mitochondrial membrane protease-like protein, which is required for processing cytochromes inside mitochondria. Numerous studies reported that IMMP2L may act as risk factor for neurological disease (Gimelli et al., 2014) and it has been associated with breast cancer (Mosca et al., 2010), although its role is not clear. Interestingly, we can see that ESR1 appears in both the lists, which provides an indication of its increasing role in responding to the estrogen stimulation.

Considering cancer related genes, we find very important oncogenes such as AKT, a serine/threonine-specific protein kinase that plays a key role in multiple cellular processes such as glucose metabolism, apoptosis, cell proliferation, transcription, and cell migration (Paplomata and O'Regan, 2014), and PTK2, a focal adhesion-associated protein kinase involved in cellular adhesion and spreading processes (Sulzmaier et al., 2014). It has been shown that when PTK2 is blocked, breast cancer cells became less metastatic due to decreased mobility (Sulzmaier et al., 2014). Another interesting gene is PTPRK, a member of the protein tyrosine phosphatase (PTP) family, known to be a signaling molecule that regulate a variety of cellular processes including cell growth, differentiation, mitotic cycle, and oncogenic transformation (Sun et al., 2013). Moreover, FYN is a Proto-oncogene tyrosine-protein kinase of the Src family of kinases, typically associated with T-cell and neuronal signaling in development and normal cell physiology. Disruptions FYN signaling pathways often have implications in the formation of a variety of cancers (Saito et al., 2010).

From the pathways point of view, after E2 stimulation, we can see that enriched processes are *signal transduction*, in which AKT1 CSF1R ESR1 MAP2K6 PTPRK RPS6KB1 are involved (*p* < 8.1e^−3^), and *platelet activation*, in regards of the genes AKT1, FYN, DGKB, and DGKI (*p* < 2.9e^−4^).

More generally, the analysis of the histograms shows that after the stimulation there are more breast cancer related genes, able to accumulate more mutations, as it emerges from comparing left and right panels (please note that they have different scales). After the stimulation there are more connections in the layer representing cancer mutations, according to the GO functional annotation network. This is interesting because it shows how mutated genes can be activated by the E2 stimulation, including cell cycle related genes, such as SYNE1, NEK7, and RPS6KB1 (Li et al., 2015).

**Figure 6:**
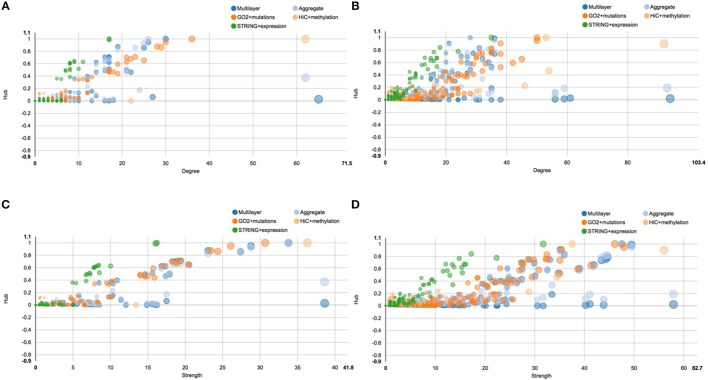
**Scatter plots of hub centrality**. This figure shows the trend of the hub centrality against node degree and strength, before and after E2 stimulation. All scatter plots represent each single layer separately, the aggregate layer (the layer with all the nodes represented in the other layers) and the whole multi-layer model. In particular: **(A)** shows the scatter plot of hub centrality against node degree before the stimulation; **(B)** shows the scatter plot of hub centrality against node degree after the stimulation; **(C)** shows the scatter plot of hub centrality against node strength before the stimulation; **(D)** shows the scatter plot of hub centrality against node strength after the stimulation.

A similar situation is described by the centrality plots reported in Figure 6, which represent the hub centrality against node degree and strength, before and after E2 stimulation. All the scatter plots represent each single layer separately, the aggregate layer (the layer with all the nodes represented in the other layers) and the whole multi-layer model. In particular, Figure 6A shows the scatter plot of hub centrality against node degree before the stimulation, which should be compared with Figure 6B, showing the scatter plot of hub centrality against node degree after the stimulation. On the other hand, Figure 6C shows the scatter plot of hub centrality against node strength before the stimulation and should be compared with Figure 6D, showing the scatter plot of hub centrality against node strength after the stimulation.

Similarly to what happens in Figure 5, also in this case we can clearly see that the multi-layer graph become more complex after the E2 stimulation, in particular due to the layer representing cancer mutations. Following the orange dots in the scatter plots, we can see that many cancer related genes turn into the network, carrying a lot of different mutations. Moreover, we can see that the chromosome conformation layer becomes more connected, in function both of the degree and strength of the node, as discussed above. On the other hand, this do not correspond to an increase of the protein-protein interactions at the gene expression level, which shows a different trend compared to the other two layers. For a full description of the centrality measures computed with MuxViz on the 0 and 4 h multi-layer models see Supplementary Tables 1, 2, respectively.

**Figure 7:**
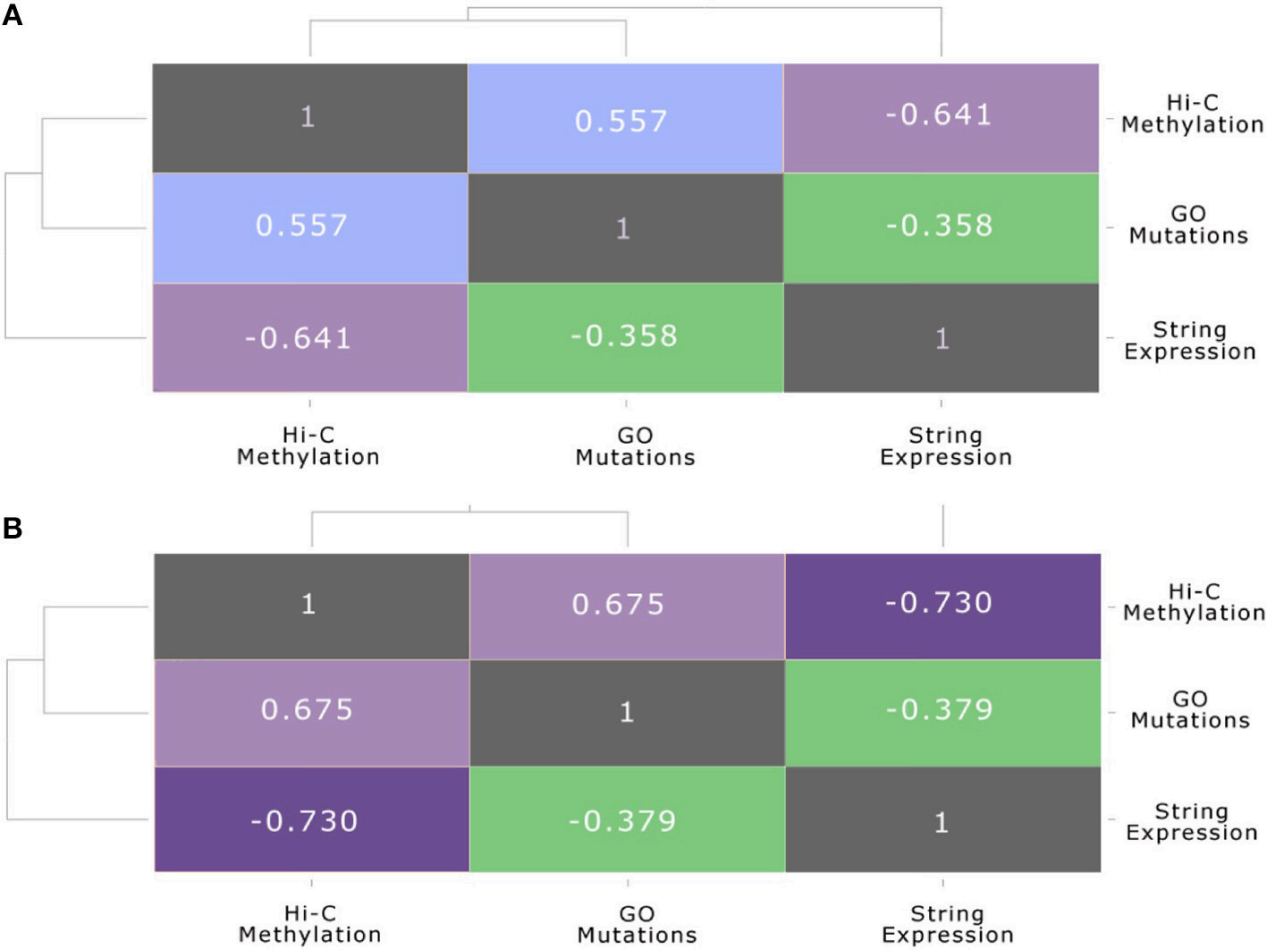
**Multi-layer correlation**. This figure shows the correlation between each pair of layers, before and after the stimulation, calculated using the Spearman correlation coefficient between the strength of nodes and their counterparts in the other layers. In particular: **(A)** shows the inter-layer Pearson correlation (Spearman correlation is reported in brackets) before the stimulation: **(B)** shows the inter-layer Pearson correlation (Spearman correlation is reported in brackets) after the stimulation.

The same association between the functional gene level and the chromosome conformation level, as well as the differences of these two layers with the protein-protein interaction level, can be also seen in Figure 7. These plots show the correlation between each pair of layers, before and after the stimulation, calculated using the Spearman correlation, which uses ranks to compare non-linear relationship, between the node strength of each level and their counterparts in the other layers. In particular, Figure 7A shows the inter-layer Spearman correlation before the stimulation, while Figure 7B shows the inter-layer Spearman correlation after stimulation.

This multi-layer correlation is very important, since it highlights the progression of the E2 signal from one layer to the other. In particular, we can see that after the stimulation the folding of the genome changes, due to a general condensation of the chromatin, which brings many genes in the neighborhood of the seed gene ESR1, although the impact on gene expression is still limited, as reported also in previous works (Cicatiello et al., 2010; Rao et al., 2014). On the other hand, the anti-correlated profile of methylation and gene expression appears quite clearly, also presenting an increasing trend after E2 stimulation. It is predictable that many hours after the E2 stimulation the impact of these genome rearrangements will be more effective in modifying the gene expression and the metabolic profile of cells.

The power of this multi-layer approach is in the capability of providing a global view of cancer evolution from a multi-omic point of view. For this reason, we further investigated the achieved results, in order to verify the type of mutations in the genes of the two graphs, before and after the stimulation. In particular, we are interested in verifying if accumulated mutations are *drivers* or *passengers* and their correlation with the genome conformation.

Moreover, we annotated all the SNPs through a score describing their pathogenic potential. Although there are many computational prediction algorithms capable of analysing the functional consequences of SNPs, we employed the Functional Analysis through Hidden Markov Models (FATHMM) algorithm (Shihab et al., 2014), a sequence-based method that combines evolutionary conservation in homologous sequences with pathogenicity weights, representing the overall tolerance of proteins to mutations. We adopted this solution because the FATHMM algorithm has a cancer specific weighting schema, which substantially improves its predictive performances in relation to other tools.

Table 1 provides a statistical description of the mutations included in the model, before and after the stimulation. In particular, according to the FATHMM algorithm, mutations with a score above the 0.7 threshold are classified as drivers, while mutations below this threshold are considered passengers. As shown in Table 1, before the stimulation our multi-layer model consists of 114 genes, potentially carrying 15,611 mutations, 10,952 of which having pathogenic potential. After the stimulation, the number of genes increases to 353 (due to the changes in the chromosome conformation), and the total number of potential mutations reaches 63,689, of which 44,678 with a pathogenic impact.

**Table 1:** **Statistical description of SNPs pathogenicity before and after E2 stimulation**.

**Time (hours)**	**Type**	**Number of SNPs**	**Number of SNPs in Metabolic Pathways**	**FATHMM Mean**	**FATHMM Median**	**FATHMM Variance**
0	Passenger	4659	422	0.190490	0.16433	0.019447
0	Driver	10952	869	0.956110	0.97762	0.003345
0	All	15611	1662	0.834172	0.96973	0.07913
4	Passenger	19011	1848	0.210717	0.21949	0.018901
4	Driver	44678	5707	0.965158	0.98166	0.002377
4	All	63689	9693	0.881392	0.974510	0.057060

SNPs are annotated as DRIVER or PASSENGER according to their FATHMM score (threshold = 0.7). The third column shows the number of SNPs for each class, while the fourth column reports the number of metabolic SNPs for each class. In the following columns descriptive statistics about the FATHMM scores of the identified SNPs are reported.

Considering that metabolic activities are altered in cancer cells and that these alterations support the acquisition and maintenance of malignant properties (DeBerardinis and Chandel, 2016), we also analyzed how many SNPs belong to metabolic genes. Using HumanMine (Smith et al., 2012), we identified genes and mutations involved in metabolic pathways: as reported in Table 1, after the stimulation we can see that the number of SNPs playing a role in metabolic processes increases, both considering driver and passenger mutations. Moreover, looking at the mean and median values of the scores distribution, we can see that there is an average increase of the pathogenic potential after the stimulation, taking into account both driver and passenger mutations.

In order to test the significance of the changes occurred after the E2 stimulation, we tested the enrichment of SNPs associated to breast cancer using a hypergeometric test. In particular, we considered all the human genes as reference and the genes belonging to the graph as sample, using the COSMIC database to identify SNPs related to breast cancer. As expected, considering that the seed gene ESR1 is related to breast cancer, both at 0 and 4 h the graphs are enriched in SNPs associated to breast cancer, with *p* < 9.3e^−20^ and *p* < 1.74e^−27^ respectively. It is worthy to note that after the E2 stimulation the enrichment is more evident than at the beginning of the experiment, which highlights a correlation between spatially related genes and functional related genes.

## Discussion

Multi-omic approaches can provide very much desirable progresses in non-invasive diagnostic methods, to enable early diagnosis, in pre- and post-operative staging, and to assist in selecting the most suitable therapeutic methods and post-treatment decisions. In this sense, chromosome conformation can help in identifying novel biomarkers (Michailidou et al., 2013), which are absent in healthy persons and present in cancer, especially at early developmental stage of the disease, in order to use them in screening tests (Merelli et al., 2013a).

To improve our understanding of the disease progression and to assist biologists in the interpretation of the results, mathematical models can be designed to encompass the modifications of the 3D genome conformation in cancer cells, in order to predict the survival probabilities of patients with different genetic expression and epigenetic patterns. In particular, using multi-omic data, it will be possible to select the best medical treatment for each patient and follow the results of drugs administration with more awareness. Moreover, survival probabilities generated with these models will be useful to identify the presence of hidden markers currently not considered, proving a full implementation of the translational medicine paradigm.

Although many different approaches can be used for multi-omic data integration, some basic assumption should be made a priori. First, we must decide whether or not to use graphs for modeling the interactions among variables (Risca and Greenleaf, 2015). Our choice was to use a graph-based approach in order to exploit the power of the topological description provided by Hi-C data, completing this information with other scalar characterization of vertices. Although this approach is not applicable in all cases, mainly because Hi-C data are not yet widely available, we think that this representation can be very useful, in particular when it is possible to include it in a multi-level model like the one presented.

The second criterion is whether the approach should be Bayesian (Korbel and Lee, 2013) or not, which depends on the possibility of creating a priori reasonable assumptions about the data probability distribution, parametric or non-parametric, and to compute the posterior probability distribution making use of the Bayes' rule as data becomes available. Although this is a very interesting option, our knowledge of the multi-level interactions, which can occur in co-expression, co-regulation and co-localization, are still very difficult to model.

Although modeling a priori can be prohibitive, we can suggest the application of some modeling techniques to interpret, a posteriori, multi-layer representations like the one we presented in this work. The idea is to go beyond the descriptive analysis (such as node degree, node strength, walks, paths, distances, centrality measures, cluster coefficients, inter-layer diagnostics, communicability; Estrada and Gómez-Gardeñes, 2014), by creating randomized network ensembles that can help in the comprehension of the network model.

More precisely, approaches used for knowledge representation can be employed to stochastically model multi-omic data, such as the Exponential-family Random Graph Models (ERGM) (Hunter et al., 2008), scale-free network models (Barabási–Albert model) (Albert and Barabási, 2002) and Multi-layer graph entropy (Bianconi, 2007). In particular, ERGM are extremely useful for network analysis, since they allow the creation of probability distributions by which some peculiarities of the graph can be extrapolated. The use of this approach will allow the probabilities to be tested, for example, to verify if edges are functions of specific genomic features or to measure the significance of having edges in relation to the specific properties of nodes. Considering that the organization of the chromatin inside the nucleus can be itself a disease biomarker, since specific reorganizations of the genome can be used for the early identification of some diseases, scale-free approaches to network modeling will be extremely useful to capture the “fold of folds” pattern of the genome. Moreover, clustering approaches, ontology annotations, and deep learning techniques can be applied to multi-layer graphs, in order to capture the impact of gene co-localization on biochemical pathways' regulation, in a systems biology perspective.

**Figure 8:**
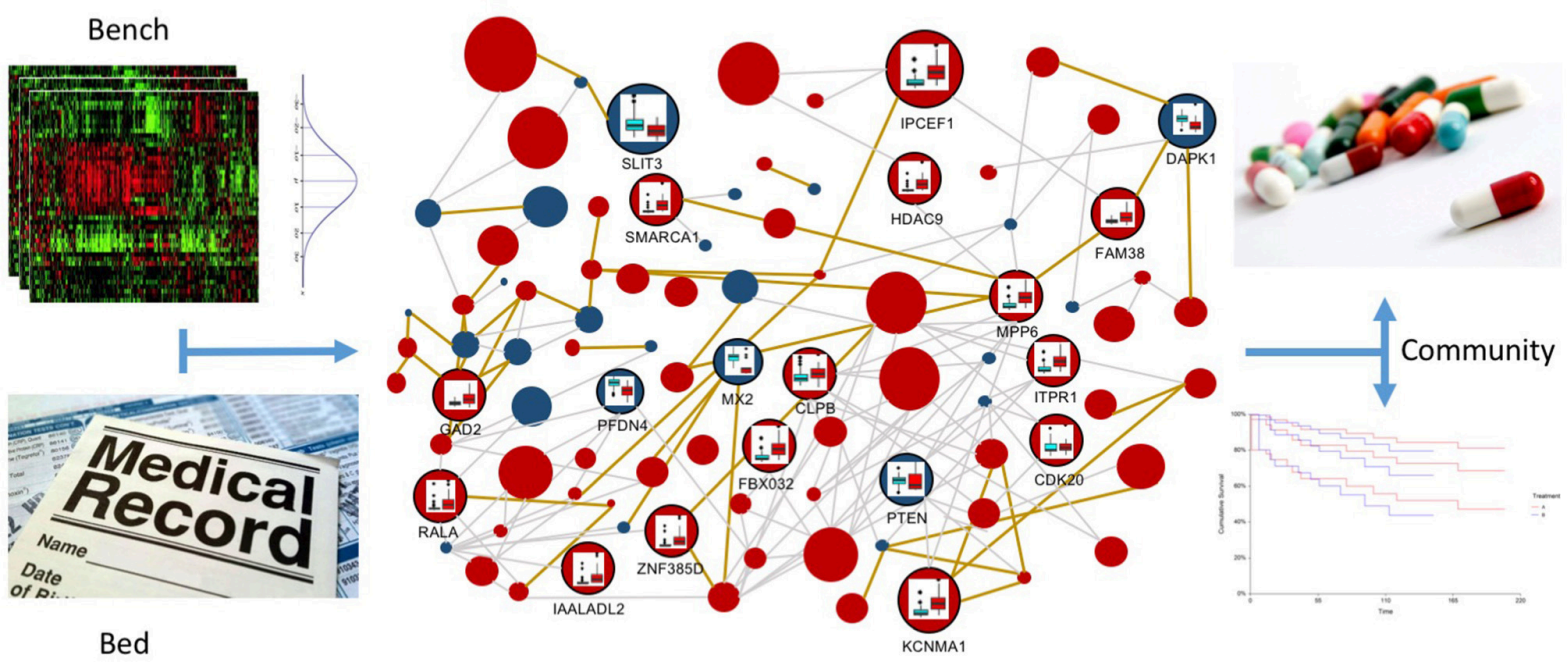
**Multi-layer based representation of multi-omic data to implement the translational medicine model**. In the upper-left panel the “bench” part of the model is represented, in which statistics are used to define confidence intervals for multi-omic data, in order to classify physiological and pathological behaviors. This information is the core of the multi-layer graph representation, since it allows to define confidence intervals for vertices and edges, which describe normal variations inside the experiments and variations that are symptomatic of the different disease stages. In the lower-left part of the Figure, the “bed” part of the model is represented, in which the omic information of the patient are reported in the electronic health record. This data will be integrated in the multi-layer model, in order to profile and interpret the specific patient, taking into account all the available multi-omics. In the right part of the figure the “community” part of the translational medicine model is represented, where inferred information is used to improve the current decision trees for treatment identification and to perform better predictions about patient survival possibilities.

The combination of integration models, such as the one presented here, with variance analysis on expression and epigenetic experiments, achieved through the integration of publicly available databases, as well as on the topology of the networks, can be the winning strategy for translational medicine (see Figure 8). It is very important to establish which variations are normal inside a specific experiment and which variations are able to distinguish between normal and pathological samples (the bench part of the translational medicine schema). Moreover, variations in the omic profiles can characterize the progression of the disease during time, describing its temporal evolution according to the provided therapies. These confidence intervals will be then compared with the patient data, in order to better characterize the pathology (the bed part of the translational medicine schema). In this context, resources like METABRIC (Curtis et al., 2012) (Molecular Taxonomy of Breast Cancer International Consortium) will be very important to identify cancer mutations able to drive genome conformational changes.

Providing a better characterization of the disease, we will achieve better treatment definitions. Expanding this idea, it will be possible to use confidence intervals for the diagnosis of disease co-morbidity by identifying specific patterns in multi-omic variability. At the same time, this multi-omic approach will be useful to improve the prediction of the survival possibility of patients, as the integration of more information about the disease can make estimators such as the Kaplan-Meier curve more precise.

## Conclusions

It is now clear that cancer is a multi-step process, resulting from the accumulation of both genetic and epigenetic alterations of the genome. Gene mutations and epigenetic modifications have been initially viewed as two separate mechanisms participating in carcinogenesis. This is the reason why the study of tumors should be performed using different profiling strategies, including mutation analyses, phylogenetic trees, copy number variations, DNA methylations, histone modifications, transcriptomic experiments and chromatin conformation capture technologies, which may collectively be defined as *omics*. Through multi-omic analysis, researchers want to identify genes and pathways deregulated in cancer and to reveal biomarkers that may be useful for the detection and the management of the disease. Such analysis will enhance our understanding of the biology of cancer, leading to the discovery of novel diagnostic, prognostic, and therapeutic approaches that will ultimately improve the disease outcomes. An integrative model of these omic data will improve the predictive power of survival curves, allowing the definition of novel biomarker, and will provide novel multi-omic based guidelines for cancer, favoring the identification of the best treatment for each patient.

In this work we presented an approach for multi-omic data modeling using multi-layer networks, which allows the integration of graph-based representations and scalar genomic data. We tested this framework for the analysis of multi-omic data from the MCF-7 cell line, a well-known breast cancer model. At first, our chromatin conformation data analysis revealed that gene-rich genomic regions tend to interact more with each other after E2 stimulation. Second, we were able to show correlations between changes in the inter-chromosomal conformation and other omic actors, such as mutation profiles and variations of the epigenetics patterns. Moreover, using our model, we were able to characterize the accumulation of driver and passenger mutations in breast cancer, allowing a better description of the damage spreading during the evolution of the disease. This mutation enrichment can be also characterized focusing on specific pathway, as represented by the GO-based network, looking for example at metabolic processes that are so important for cancer development.

This is in line with the idea that the organization of the DNA in the nucleus represents an integrating factor for many omic signals. Although challenges about the comprehension of 3C data must still be addressed, such as the management of genetic variants in order to distinguish the conformations of the two homologous chromosomes, the possibility of studying long-range DNA interactions opens new perspectives for cancer research. Researches about genome 3D organization, and related epigenetic patterns in the nucleus, will be very important to understand cancer progression, providing novel biomarkers to identify the early onset of the disease. This can be achieved by integrating variance analyses of multi-omic data in this model, relying on statistics about physiological and pathological molecular patterns, improving both treatment decision trees and survival estimators, such as the Kaplan-Meier curves.

In our vision, different cancer omics—mutations, chromatin conformation, epigenetics, gene expression, variations in the metabolic pathways—will be calibrated performing a quality assessment of the data on the basis of the mutual consistence, in order to achieve a model for interpreting patient data. In other words, omic data from patients should be considered in terms of variances, which will be used to take decisions by comparing results with well-established network models of cancer, describing the temporal evolution of the disease along with the appropriate therapies. The open problem is to create suitable genomic and bioinformatic tools for these clinical applications.

## Author contributions

PL and IM conceived the work. FT and IM implemented the multi-layer model. MA, LM, and PL supported and supervised the work. IM and FT wrote the paper.

## Funding

This work has been supported by the Italian Ministry of Education and Research (MIUR) through the Flagship (PB05) InterOmics, the EC-FP7 innovation project “MIMOMICS” (no. 305280), and the EC-FP7 strep project “REPARA” (no. 609666).

### Conflict of interest statement

The authors declare that the research was conducted in the absence of any commercial or financial relationships that could be construed as a potential conflict of interest.

## References

[B1] Albert R. Barabási A. L. (2002). Statistical mechanics of complex networks. Rev. Mod. Phys. 74:47 10.1103/RevModPhys.74.47

[B2] Babaei S. Mahfouz A. Hulsman M. Lelieveldt B. P. de Ridder J. Reinders M. (2015). Hi-C chromatin interaction networks predict co-expression in the mouse cortex. PLoS Comput. Biol. 11:e1004221. 10.1371/journal.pcbi.1004221 25965262 PMC4429121

[B3] Babu D. Fullwood M. J. (2015). 3D genome organization in health and disease: emerging opportunities in cancer translational medicine. Nucleus 6, 382–393. 10.1080/19491034.2015.1106676 26553406 PMC4915485

[B4] Barutcu A. R. Fritz A. J. Zaidi S. K. van Wijnen A. J. Lian J. B. Stein J. L.. (2016). C-ing the genome: a compendium of chromosome conformation capture methods to study higher-order chromatin organization. J. Cell Physiol. 231, 31–35. 10.1002/jcp.25062 26059817 PMC4586368

[B5] Bianconi G. (2007). The entropy of randomized network ensembles. Europhys. Lett. 81:28005 10.1209/0295-5075/81/28005

[B6] Biasco L. Ambrosi A. Pellin D. Bartholomae C. Brigida I. Roncarolo M. G.. (2011). Integration profile of retroviral vector in gene therapy treated patients is cell-specific according to gene expression and chromatin conformation of target cell. EMBO Mol. Med. 3, 89–101. 10.1002/emmm.201000108 21243617 PMC3060339

[B7] Cicatiello L. Mutarelli M. Grober O. M. Paris O. Ferraro L. Ravo M.. (2010). Estrogen receptor α controls a gene network in luminal-like breast cancer cells comprising multiple transcription factors and microRNAs. Am. J. Pathol. 176, 2113–2130. 10.2353/ajpath.2010.090837 20348243 PMC2861078

[B8] Coppedè F. Lopomo A. Spisni R. Migliore L. (2014). Genetic and epigenetic biomarkers for diagnosis, prognosis and treatment of colorectal cancer. World J. Gastroenterol. 20, 943–956. 10.3748/wjg.v20.i4.943 24574767 PMC3921546

[B9] Curtis C. Shah S. P. Chin S. F. Turashvili G. Rueda O. M. Dunning M. J.. (2012). The genomic and transcriptomic architecture of 2,000 breast tumours reveals novel subgroups. Nature 486, 346–352. 10.1038/nature10983 22522925 PMC3440846

[B10] De Domenico M. Porter M. A. Arenas A. (2015). MuxViz: a tool for multilayer analysis and visualization of networks. J. Complex Netw. 3, 159–176. 10.1093/comnet/cnu038

[B11] De Domenico M. Solé-Ribalta A. Cozzo E. Kivelä M. Moreno Y. Porter M. A. (2013). Mathematical formulation of multilayer networks. Phys. Rev. X 3, 041022 10.1103/physrevx.3.041022

[B12] DeBerardinis R. J. Chandel N. S. (2016). Fundamentals of cancer metabolism. Sci. Adv. 2:e1600200. 10.1126/sciadv.1600200 27386546 PMC4928883

[B13] Dong X. Li C. Chen Y. Ding G. Li Y. (2010). Human transcriptional interactome of chromatin contribute to gene co-expression. BMC Genomics 11:1. 10.1186/1471-2164-11-704 21156067 PMC3053592

[B14] Du M. Tillmans L. Gao J. Gao P. Yuan T. Dittmar R. L.. (2016). Chromatin interactions and candidate genes at ten prostate cancer risk loci. Sci. Rep. 6:23202. 10.1038/srep23202 26979803 PMC4793270

[B15] ENCODE Project Consortium (2012). An integrated encyclopedia of DNA elements in the human genome. Nature 489, 57–74. 10.1038/nature11247 22955616 PMC3439153

[B16] Estrada E. Gómez-Gardeñes J. (2014). Communicability reveals a transition to coordinated behavior in multiplex networks. Phys. Rev. E 89:042819. 24827305 10.1103/PhysRevE.89.042819

[B17] Forbes S. A. Beare D. Gunasekaran P. Leung K. Bindal N. Boutselakis H.. (2015). COSMIC: exploring the world's knowledge of somatic mutations in human cancer. Nucleic Acids Res. 43, D805–D811. 10.1093/nar/gku1075 25355519 PMC4383913

[B18] Gerstein M. B. Kundaje A. Hariharan M. Landt S. G. Yan K. K. Cheng C.. (2012). Architecture of the human regulatory network derived from ENCODE data. Nature 489, 91–100. 10.1038/nature11245 22955619 PMC4154057

[B19] Gimelli S. Capra V. Di Rocco M. Leoni M. Mirabelli-Badenier M. Schiaffino M. C.. (2014). Interstitial 7q31. 1 copy number variations disrupting IMMP2L gene are associated with a wide spectrum of neurodevelopmental disorders. Mol. Cytogenet. 7, 1. 10.1186/s13039-014-0054-y 25478008 PMC4255718

[B20] Heinz S. Benner C. Spann N. Bertolino E. Lin Y. C. Laslo P.. (2010). Simple combinations of lineage-determining transcription factors prime cis-regulatory elements required for macrophage and B cell identities. Mol. Cell 38, 576–589. 10.1016/j.molcel.2010.05.004 20513432 PMC2898526

[B21] Homouz D. Kudlicki A. S. (2013). The 3D organization of the yeast genome correlates with co-expression and reflects functional relations between genes. PLoS ONE 8:e54699. 10.1371/journal.pone.0054699 23382942 PMC3561378

[B22] Horike S. I. Cai S. Miyano M. Cheng J. F. Kohwi-Shigematsu T. (2005). Loss of silent-chromatin looping and impaired imprinting of DLX5 in Rett syndrome. Nat. Genet. 37, 31–40. 10.1038/ng1491 15608638

[B23] Hsu P. Y. Hsu H. K. Singer G. A. Yan P. S. Rodriguez B. A. Liu J. C.. (2010). Estrogen-mediated epigenetic repression of large chromosomal regions through DNA looping. Genome Res. 20, 733–744. 10.1101/gr.101923.109 20442245 PMC2877570

[B24] Hunter D. R. Handcock M. S. Butts C. T. Goodreau S. M. Morris M. (2008). ergm: a package to fit, simulate and diagnose exponential-family models for networks. J. Stat. Softw. 24, nihpa54860. 10.18637/jss.v024.i03 19756229 PMC2743438

[B25] Jäger R. Migliorini G. Henrion M. Kandaswamy R. Speedy H. E. Heindl A.. (2015). Capture Hi-C identifies the chromatin interactome of colorectal cancer risk loci. Nat. Commun. 6:6178. 10.1038/ncomms7178 25695508 PMC4346635

[B26] Jensen L. J. Kuhn M. Stark M. Chaffron S. Creevey C. Muller J.. (2009). STRING 8—a global view on proteins and their functional interactions in 630 organisms. Nucleic Acids Res. 37, D412–D416. 10.1093/nar/gkn760 18940858 PMC2686466

[B27] Jin F. Li Y. Dixon J. R. Selvaraj S. Ye Z. Lee A. Y.. (2013). A high-resolution map of the three-dimensional chromatin interactome in human cells. Nature 503, 290–294. 10.1038/nature12644 24141950 PMC3838900

[B28] Kalhor R. Tjong H. Jayathilaka N. Alber F. Chen L. (2012). Genome architectures revealed by tethered chromosome conformation capture and population-based modeling. Nat. Biotechnol. 30, 90–98. 10.1038/nbt.2057 22198700 PMC3782096

[B29] Kel I. Chang Z. Galluccio N. Romeo M. Beretta S. Diomede L.. (2016). SPIRE, a modular pipeline for eQTL analysis of RNA-Seq data, reveals a regulatory hotspot controlling miRNA expression in *C. elegans*. Mol. BioSyst. 12, 3447–3458. 10.1039/C6MB00453A 27722582

[B30] Keune W. J. Sims A. H. Jones D. R. Bultsma Y. Lynch J. T. Jirström K.. (2013). Low PIP4K2B expression in human breast tumors correlates with reduced patient survival: a role for PIP4K2B in the regulation of E-cadherin expression. Cancer Res. 73, 6913–6925. 10.1158/0008-5472.CAN-13-0424 24127122 PMC5321494

[B31] Kivelä M. Arenas A. Barthelemy M. Gleeson J. P. Moreno Y. Porter M. A. (2014). Multilayer networks. J. Complex Netw. 2, 203–271. 10.1093/comnet/cnu01618276399

[B32] Korbel J. O. Lee C. (2013). Genome assembly and haplotyping with Hi-C. Nat. Biotechnol. 31, 1099–1101. 10.1038/nbt.2764 24316648

[B33] Lan X. Witt H. Katsumura K. Ye Z. Wang Q. Bresnick E. H.. (2012). Integration of Hi-C and ChIP-seq data reveals distinct types of chromatin linkages. Nucleic Acids Res. 40, 7690–7704. 10.1093/nar/gks501 22675074 PMC3439894

[B34] Li S. Zhu Y. Ma C. Qiu Z. Zhang X. Kang Z.. (2015). Downregulation of EphA5 by promoter methylation in human prostate cancer. BMC Cancer 15:1. 10.1186/s12885-015-1025-3 25609195 PMC4307617

[B35] Lieberman-Aiden E. Van Berkum N. L. Williams L. Imakaev M. Ragoczy T. Telling A.. (2009). Comprehensive mapping of long-range interactions reveals folding principles of the human genome. Science 326, 289–293. 10.1126/science.1181369 19815776 PMC2858594

[B36] Lin J. E. Li P. Snook A. E. Schulz S. Dasgupta A. Hyslop T. M.. (2010). The hormone receptor GUCY2C suppresses intestinal tumor formation by inhibiting AKT signaling. Gastroenterology 138, 241–254. 10.1053/j.gastro.2009.08.064 19737566 PMC2813361

[B37] Maffini M. V. Soto A. M. Calabro J. M. Ucci A. A. Sonnenschein C. (2004). The stroma as a crucial target in rat mammary gland carcinogenesis. J. Cell Sci. 117, 1495–1502. 10.1242/jcs.01000 14996910

[B38] Meaburn K. J. Gudla P. R. Khan S. Lockett S. J. Misteli T. (2009). Disease-specific gene repositioning in breast cancer. J. Cell Biol. 187, 801–812. 10.1083/jcb.200909127 19995938 PMC2806312

[B39] Merelli I. Calabria A. Cozzi P. Viti F. Mosca E. Milanesi L. (2013a). SNPranker 2.0: a gene-centric data mining tool for diseases associated SNP prioritization in GWAS. BMC Bioinformatics 14:S9. 10.1186/1471-2105-14-S1-S9 23369106 PMC3548692

[B40] Merelli I. Guffanti A. Fabbri M. Cocito A. Furia L. Grazini U.. (2010). RSSsite: a reference database and prediction tool for the identification of cryptic Recombination Signal Sequences in human and murine genomes. Nucleic Acids Res. 38(Suppl. 2), W262–W267. 10.1093/nar/gkq391 20478831 PMC2896083

[B41] Merelli I. Liò P. Milanesi L. (2013b). NuChart: an R package to study gene spatial neighbourhoods with multi-omics annotations. PLoS ONE 8:e75146. 10.1371/journal.pone.0075146 24069388 PMC3777921

[B42] Merelli I. Tordini F. Drocco M. Aldinucci M. Liò P. Milanesi L. (2015). Integrating multi-omic features exploiting Chromosome Conformation Capture data. Front. Genet. 6:40. 10.3389/fgene.2015.00040 25717338 PMC4324155

[B43] Michailidou K. Hall P. Gonzalez-Neira A. Ghoussaini M. Dennis J. Milne R. L.. (2013). Large-scale genotyping identifies 41 new loci associated with breast cancer risk. Nat. Genet. 45, 353–361. 10.1038/ng.2563 23535729 PMC3771688

[B44] Mosca E. Alfieri R. Merelli I. Viti F. Calabria A. Milanesi L. (2010). A multilevel data integration resource for breast cancer study. BMC Syst. Biol. 4:1. 10.1186/1752-0509-4-76 20525248 PMC2900226

[B45] Mourad R. Hsu P. Y. Juan L. Shen C. Koneru P. Lin H.. (2014). Estrogen induces global reorganization of chromatin structure in human breast cancer cells. PLoS ONE 9:e113354. 10.1371/journal.pone.0113354 25470140 PMC4255042

[B46] Papantonis A. Cook P. R. (2013). Transcription factories: genome organization and gene regulation. Chem. Rev. 113, 8683–8705. 10.1021/cr300513p 23597155

[B47] Paplomata E. O'Regan R. (2014). The PI3K/AKT/mTOR pathway in breast cancer: targets, trials and biomarkers. Ther. Adv. Med. Oncol. 6, 154–166. 10.1177/1758834014530023 25057302 PMC4107712

[B48] Peng J. Li H. Jiang Q. Wang Y. Chen J. (2014). An integrative approach for measuring semantic similarities using gene ontology. BMC Syst. Biol. 8:S8. 10.1186/1752-0509-8-S5-S8 25559943 PMC4305987

[B49] Petrov A. Allinne J. Pirozhkova I. Laoudj D. Lipinski M. Vassetzky Y. S. (2008). A nuclear matrix attachment site in the 4q35 locus has an enhancer-blocking activity *in vivo*: implications for the facio-scapulo-humeral dystrophy. Genome Res. 18, 39–45. 10.1101/gr.6620908 18032730 PMC2134777

[B50] Putnik M. Zhao C. Gustafsson J. Å. Dahlman-Wright K. (2012). Global identification of genes regulated by estrogen signaling and demethylation in MCF-7 breast cancer cells. Biochem. Biophys. Res. Commun. 426, 26–32. 10.1016/j.bbrc.2012.08.007 22902638

[B51] Rao S. S. Huntley M. H. Durand N. C. Stamenova E. K. Bochkov I. D. Robinson J. T.. (2014). A 3D map of the human genome at kilobase resolution reveals principles of chromatin looping. Cell 159, 1665–1680. 10.1016/j.cell.2014.11.021 25497547 PMC5635824

[B52] Rickman D. S. Soong T. D. Moss B. Mosquera J. M. Dlabal J. Terry S.. (2012). Oncogene-mediated alterations in chromatin conformation. Proc. Natl. Acad. Sci.U.S.A. 109, 9083–9088. 10.1073/pnas.1112570109 22615383 PMC3384175

[B53] Risca V. I. Greenleaf W. J. (2015). Unraveling the 3D genome: genomics tools for multiscale exploration. Trends Genet. 31, 357–372. 10.1016/j.tig.2015.03.010 25887733 PMC4490074

[B54] Ross-Innes C. S. Brown G. D. Carroll J. S. (2011). A co-ordinated interaction between CTCF and ER in breast cancer cells. BMC Genomics 12:593. 10.1186/1471-2164-12-593 22142239 PMC3248577

[B55] Saito Y. D. Jensen A. R. Salgia R. Posadas E. M. (2010). Fyn : a novel molecular target in cancer. Cancer 116, 1629–1637. 10.1002/cncr.24879 20151426 PMC2847065

[B56] Schoenfelder S. Sexton T. Chakalova L. Cope N. F. Horton A. Andrews S.. (2010). Preferential associations between co-regulated genes reveal a transcriptional interactome in erythroid cells. Nat. Genet. 42, 53–61. 10.1038/ng.496 20010836 PMC3237402

[B57] Shavit Y. (2013). CytoHiC: a cytoscape plugin for visual comparison of Hi-C networks. Bioinformatics 29, 1206–1207. 10.1093/bioinformatics/btt120 23508968

[B58] Shen Y. Yue F. McCleary D. F. Ye Z. Edsall L. Kuan S.. (2012). A map of the cis-regulatory sequences in the mouse genome. Nature 488, 116–120. 10.1038/nature11243 22763441 PMC4041622

[B59] Shihab H. A. Gough J. Mort M. Cooper D. N. Day I. N. Gaunt T. R. (2014). Ranking non-synonymous single nucleotide polymorphisms based on disease concepts. Hum. Genomics 8, 1. 10.1186/1479-7364-8-11 24980617 PMC4083756

[B60] Smith R. N. Aleksic J. Butano D. Carr A. Contrino S. Hu F.. (2012). InterMine: a flexible data warehouse system for the integration and analysis of heterogeneous biological data. Bioinformatics 28, 3163–3165. 10.1093/bioinformatics/bts577 23023984 PMC3516146

[B61] Sulzmaier F. J. Jean C. Schlaepfer D. D. (2014). FAK in cancer: mechanistic findings and clinical applications. Nat. Rev. Cancer 14, 598–610. 10.1038/nrc3792 25098269 PMC4365862

[B62] Sun P. H. Ye L. Mason M. D. Jiang W. G. (2013). Protein tyrosine phosphatase kappa (PTPRK) is a negative regulator of adhesion and invasion of breast cancer cells, and associates with poor prognosis of breast cancer. J. Cancer Res. Clin. Oncol. 139, 1129–1139. 10.1007/s00432-013-1421-5 23552869 PMC11824379

[B63] Taberlay P. C. Achinger-Kawecka J. Lun A. T. Buske F. A. Sabir K. Gould C. M.. (2016). Three-dimensional disorganization of the cancer genome occurs coincident with long-range genetic and epigenetic alterations. Genome Res. 26, 719–731. 10.1101/gr.201517.115 27053337 PMC4889976

[B64] Vucic E. A. Thu K. L. Robison K. Rybaczyk L. A. Chari R. Alvarez C. E.. (2012). Translating cancer ‘omics’ to improved outcomes. Genome Res. 22, 188–195. 10.1101/gr.124354.111 22301133 PMC3266027

[B65] Zhang Y. Liu T. Meyer C. A. Eeckhoute J. Johnson D. S. Bernstein B. E.. (2008). Model-based analysis of ChIP-Seq (MACS). Genome Biol. 9, 1. 10.1186/gb-2008-9-9-r137 18798982 PMC2592715

[B66] Zhang Y. McCord R. P. Ho Y. J. Lajoie B. R. Hildebrand D. G. Simon A. C.. (2012). Spatial organization of the mouse genome and its role in recurrent chromosomal translocations. Cell 148, 908–921. 10.1016/j.cell.2012.02.002 22341456 PMC3320767

